# Imeglimin preserves islet β‐cell mass in Type 2 diabetic ZDF rats

**DOI:** 10.1002/edm2.193

**Published:** 2020-11-07

**Authors:** Sophie Hallakou‐Bozec, Micheline Kergoat, David E. Moller, Sébastien Bolze

**Affiliations:** ^1^ Poxel SA Lyon France; ^2^ Metabrain Research Maisons‐Alfort France

**Keywords:** animal models, islet, imeglimin, new therapies

## Abstract

**Objectives:**

Type 2 diabetes (T2D) is driven by progressive dysfunction and loss of pancreatic β‐cell mass. Imeglimin is a first‐in‐class novel drug candidate that improves glycaemia and glucose‐stimulated insulin secretion in preclinical models and patients. Given evidence that imeglimin can attenuate β‐cell dysfunction and protect β cells *in vitro*, we postulated that imeglimin could also exert longer term effects to prevent pancreatic β‐cell death and preserve functional β‐cell mass *in vivo*.

**Methods:**

Zucker diabetic fatty (ZDF) male rats were treated by oral gavage with imeglimin at a standard dose of 150 mg/kg or vehicle, twice daily for five weeks. At treatment completion, oral glucose tolerance tests were performed in fasted animals before a thorough histomorphometry and immunohistochemical analysis was conducted on pancreas tissue slices to assess cellular composition and disease status.

**Results:**

Imeglimin treatment significantly improved glucose‐stimulated insulin secretion (augmentation of the insulinogenic index) and improved glycaemia. Both basal insulinaemia and pancreatic insulin content were also increased by imeglimin. In ZDF control rats, islet structure was disordered with few β‐cells; after imeglimin treatment, islets appeared healthier with more normal morphology in association with a significant increase in insulin‐positive β‐cells. The increase in β‐cell mass was associated with a greater degree of β‐cell proliferation in the presence of reduced apoptosis. Unexpectedly, a decrease in as a α‐cell mass was also documented due to an apparent antiproliferative effect of imeglimin on this cell type.

**Conclusion:**

In male ZDF rats, chronic imeglimin treatment corrects a paramount component of type 2 diabetes progression: progressive loss of functional β‐cell mass. In addition, imeglimin may also moderate a‐cell turnover to further ameliorate hyperglycaemia. Cumulatively, these cellular effects suggest that imeglimin may provide for disease modifying effects to preserve functional β‐cell mass.

## Graphical abstract

Imeglimin is a novel drug candidate for type 2 diabetes, which has completed Phase III trials; its mechanism involves effects to augment islet β‐cell function. Imeglimin treatment of ZDF rats is shown to ameliorate glucose intolerance and increase insulinemia. Islet β‐cell mass is increased after chronic imeglimin treatment; β‐cell apoptosis is reduced, and proliferation is increased in response to imeglimin treatment.

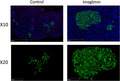

## 1 INTRODUCTION

Over the course of several decades, the worldwide prevalence of diabetes has risen dramatically.^1^, ^2^ This has largely resulted from the occurrence of obesity leading to a surge in type 2 diabetes (T2D). The pathophysiology of T2D is characterized by insulin resistance combined with a predominant impairment of insulin secretion resulting from the progressive failure of pancreatic β‐cells.^3^, ^4^ Indeed, the onset of overt T2D requires insufficient insulin secretion to compensate for insulin resistance.^3^ Notably, postmortem studies indicate that a prominent reduction in β‐cell mass also occurs in patients and appears to be a major contributor to deficient insulin secretory capacity.^4^, ^5^ Therefore, new therapeutic approaches, which encompass correction of β‐cell dysfunction and preservation of functional β‐cell mass would be highly desirable additions to our current repertoire of available therapies.^6^, ^7^

Imeglimin is a novel oral antidiabetic molecule designed for the treatment of T2D. Its novel structure and proposed mechanism of action establishes the first in a new tetrahydrotriazine class called the ‘glimins’.^8^ Three Phase III clinical trials were recently completed and a consistent absence of hypoglycaemia, excellent tolerability, and strong glycemic efficacy in T2D patients—either as a stand‐alone treatment or as an add‐on medication—were seen in multiple trials.^8^, ^9^, ^10^, ^11^

Imeglimin exhibits a dual mechanism of action—both to improve insulin action and to restore defective β‐cell function by amplifying glucose‐stimulated insulin secretion (GSIS).^12^, ^13^, ^14^ Importantly, enhanced GSIS was clearly demonstrated in patients with T2D via the use of a hyperglycaemic clamp procedure.^11^ At a molecular and cellular level, imeglimin has a prominent effect to rebalance defective mitochondrial function,^13^, ^14^, ^15^ a key aspect of T2D pathology that occurs in both islet β‐cells and other tissues.^16^, ^17^, ^18^ Interestingly, imeglimin also appears to provide for a degree of acute protection of β‐cells from high glucose‐ or pro‐inflammatory cytokine‐induced apoptosis in vitro.^12^

In light of the above findings and considering the cardinal importance of securing improved β‐cell function over time to achieve efficient and sustained management of glycemia in T2D, we performed new translational experiments to investigate whether imeglimin could mitigate the progressive loss of β‐cells in an animal model of T2D. We chose to use Zucker diabetic fatty (ZDF) rats, a genetic model of obesity‐driven T2D that is manifested by extreme hyperglycaemia and progressive, overt, apoptosis‐mediated loss of β‐cell mass.^19^ Here, we have demonstrated that a standard oral dose of imeglimin (150 mg/kg twice daily) administered for 5 weeks could result in several important effects. As seen in other animal models and in patients, an improvement in in vivo GSIS—based on the ratio of incremental increase in insulin to glucose (insulinogenic index) during glucose tolerance testing—was evident. More importantly, we demonstrated increases in β‐cell mass based on quantitation of both biochemical and histologic parameters along with a relative reduction in the proportion of apoptotic β‐cells and a reciprocal increase in proliferating β‐cells.

These findings further support our understanding of the mechanism of action of imeglimin and provide data to prompt further, longer term, clinical testing that may reveal important disease‐modifying benefits of this new class of medicines.

## 2 MATERIAL AND METHODS

### 2.1 Animal sourcing and handling

Experiments were performed on seven‐week‐old male ZDF rats purchased from Charles River Laboratories. The studies were conducted at the Metabrain Research facility (4 avenue du Président F. Mitterrand—91380 Chilly Mazarin, France) and were carried out in accordance with the European animal care guidelines (ETS 123). Animals were acclimated to the vivarium environment and trained for dosing and manipulation for 1 week before initiation of dosing in the experiment. Animals were housed in a temperature‐controlled (22 ± 2°C) room under constant humidity (50 ± 20%) and with a 12‐hour light‐dark cycle (lights on at 7:00 AM). All rats were allowed to eat normal grow diet A03 from SAFE (Scientific Animal Food and Engineering—Route de Saint Bris—89 290 AUGY—France) and drink ad libitum. The litter boxes (sterile sawdust) were changed every other day. The rats were divided into groups of 3‐4 per cage. The dimensions of the cage were 48 × 37.5 × 21 cm. General signs were observed, and only animals without any abnormal signs were included in the study.

### 2.2 Dosing and oral glucose tolerance testing

One group of animals, a total of 32 rats, were treated by oral gavage with imeglimin at the dose of 150 mg/kg twice a day or with the vehicle (Methylcellulose 0.5%) during 5 weeks beginning at 7 weeks of age. Body weight was monitored at days 1, 4, 8, 11, 15, 18, 22, 25, 29, 32, 36 and 38 after the first day of dosing. After 5 weeks of treatment, an oral glucose tolerance test (OGTT) was carried out in 3‐hour fasted rats 1 hour after the morning administration of vehicle or imeglimin. A first blood sample was collected at T0 just before the oral glucose load (2 g/kg, 5 mL/kg), and other blood samples were collected at T + 10, T + 20, T + 30, T + 60 and T + 120 min after the glucose load. All plasma samples were frozen and stored at −20°C until glucose and insulin measurements. The total area under the curve (total AUC), and the incremental AUC was calculated with the trapezoidal method.

In a second cohort of animals, a total of 8 rats were treated by oral gavage with imeglimin at the dose of 150 mg/kg twice a day or with the vehicle (Methylcellulose). At the end of 5 weeks, animals were euthanized and tissue was harvested and processed for histopathology as described below.

**FIGURE 1:**
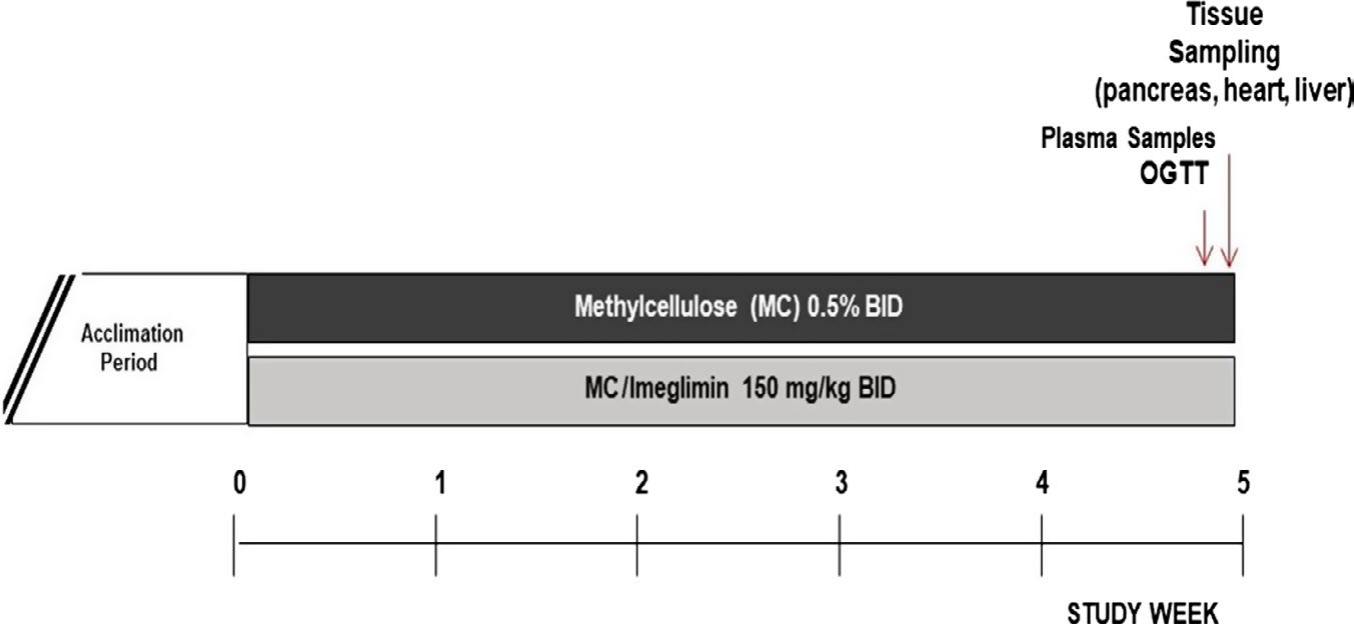
Study Design

A scheme outlining the experimental paradigm for both groups of animals is depicted in Figure 1.

### 2.3 Tissue sampling and processing

For the first group of animals, pancreas samples were collected from a subset of ZDF rats selected at random from each treated group (vehicle n = 4, imeglimin n = 10) 2 days after the OGTT and 1 hour after the morning administration of drug or vehicle. Animals were also fasted for 3 hours before being euthanized. The animals were anesthetized by IP administration of pentobarbital (50 mg/kg/4 mL) before decapitation. A fragment of pancreas was collected, weighed and stored at −20°C in vials containing a mix of acid/alcohol until insulin content measurement.

For the second group of rats, fragments of pancreas tissue were obtained from euthanized animals and were fixed in paraformaldehyde 4% and rinsed in ethanol 70%. Fixed pancreas tissue samples were embedded in paraffin. Four paraffin‐embedded pancreases from each group were used for further study. Paraffin‐embedded pancreata were serially sectioned (7 µm) throughout their length. At least 5 sections were randomly chosen every 100‐250 µm throughout the block for histology and immunohistochemistry measurements described below.

### 2.4 **Measurements of** β**‐cell mass,** β**‐cell proliferation,** β**‐cell apoptosis and** β**‐cell neogenesis**

β‐cell mass was determined by immunostaining with guinea pig anti‐insulin antibody coupled with FITC (green); cell nuclei were stained blue via labelling with DAPI (Prolong gold from Invitrogen ref. P36935). For each slice examined, the β‐cell mass (expressed as µg/mg pancreas) was calculated by quantitating the percentage of area staining positive for insulin normalized to the pancreas weight (mg). The measurement of areas was determined by using CALOPIX image analysing software.

β‐cell proliferation was estimated by measurement of Ki67 positivity of insulin‐positive cells in sections after double immunostaining with a rabbit anti‐Ki67 antibody (Abcam—Ref. ab16667) coupled with rhodamine (Fisher Scientific—Ref. 11 829 200) (red) and a polyclonal guinea pig anti‐insulin antibody coupled with FITC (green). The number of cells stained with both insulin and Ki‐67 antibodies was determined using the NDP View image analysing system software (Hamamatsu).

A double immunostaining approach with anti‐caspase 3 and insulin antibodies was performed for evaluation of apoptotic β cells. Fixed pancreatic sections were incubated with a polyclonal rabbit anti‐caspase‐3 antibody (Ozyme ref. 9664) coupled to rhodamine (red) and with a polyclonal guinea pig anti‐insulin antibody coupled with FITC (green). The number of cells stained with both insulin and caspase‐3 immunostaining was determined using the NDP View image analysing software system (Hamamatsu).

A double immunostaining approach with anti‐cytokeratin 20 and insulin antibodies was performed for evaluation of neogenesis in β‐cells. Fixed pancreatic sections were incubated with a monoclonal rabbit anti‐cytokeratin 20 (Abcam—Ref. ab76126) coupled to rhodamine (red) and with a polyclonal guinea pig anti‐insulin antibody coupled with FITC (green). Monoclonal rabbit anti‐cytokeratin 20 was used to identify pancreatic ductal cells. The number of single β‐cells, β‐cell clusters (2‐15 β‐cells) and islets (more than 15 β‐cells) identified by insulin immunostaining coupled with FITC (green) budding from the ducts was then determined using an image NDP View image analysing system software (Hamamatsu).

### 2.5 **Measurements of** α**‐cell number per islet,** α**‐cell proliferation**

Alpha cell number was estimated by counting of glucagon‐positive cells in recognizable islets present within the sections after immunostaining with a monoclonal mouse antiglucagon antibody (Abcam—Ref. ab10988) coupled with Anti IgG mouse—FITC Antibody (Eurobio—Ref. FI‐2000) (green). The total cell number in islets was estimated by counting of the nuclei stained with the fluorescent dye, DAPI. The measurement of areas was determined by using a CALOPIX image analysing software.

α‐cell proliferation was estimated by measurement of Ki67 positivity of glucagon‐positive cells in sections after double immunostaining with a rabbit anti‐Ki67 antibody (Abcam—Ref. ab16667) coupled with a goat anti rabbit IgG‐Alexa Fluor 594 (Thermo Fisher Scientific—Ref. A‐11037) (red) and a monoclonal mouse antiglucagon antibody (Abcam—Ref. ab10988) coupled with Anti IgG mouse –FITC Antibody (Eurobio—Ref. FI‐2000) (green). The number of cells stained with both glucagon and Ki‐67 immunostaining was determined using the above noted image analysing system software.

### 2.6 Statistical analysis

For all parameters, a Student's *t* test was performed to evaluate the significance of the effect of imeglimin compared to the control group with the exception of: (a) the insulinogenic index and, (b) duct‐associated cluster and single cells and basal insulinemia, where a Mann‐Whitney U test was performed because of the lack of normality. Statistical significance was set at *P* < .05.

## 3 RESULTS

### 3.1 Imeglimin improves glucose tolerance in association with enhanced insulin secretion in ZDF rats

**FIGURE 2:**
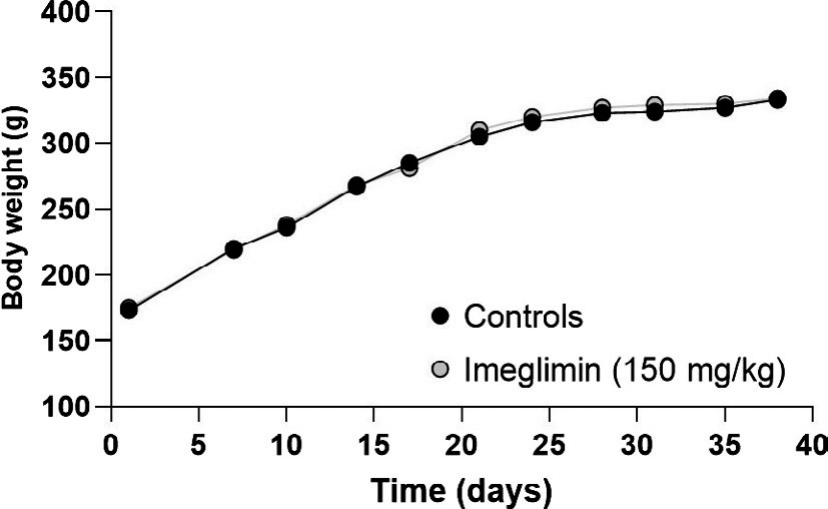
Effect of imeglimin on Body Weight after 5 wk (38 d) treatment in ZDF Rats. Mean body weights are depicted. Error bars (±SEM) are shorter than the size of the symbols and are therefore excluded from the graph shown

**FIGURE 3:**
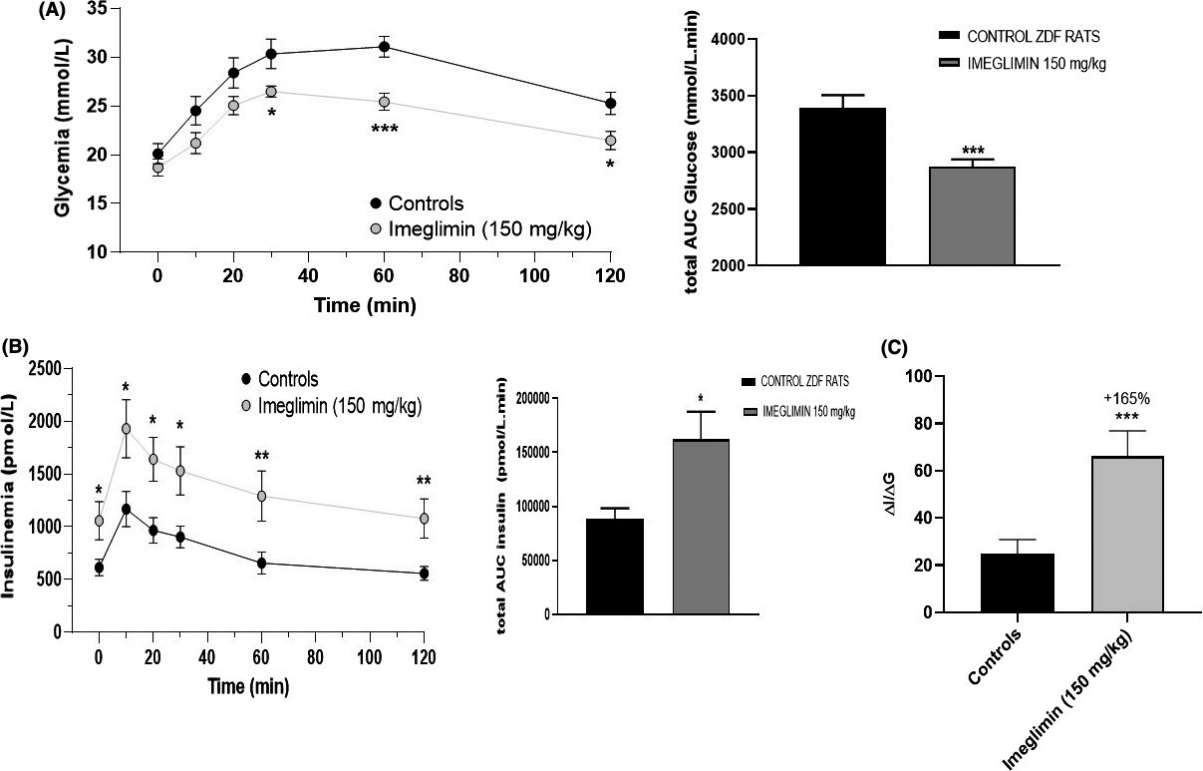
Effect of imeglimin on Glucose Tolerance and Insulin Response to Oral Glucose Loading (OGTT) in Obese Diabetic ZDF Rats. (A) Plasma glucose at indicated time points was assessed in response to oral glucose loading (2 g/kg) 1 hr after the oral administration of the vehicle (Control, n = 14) or of imeglimin at 150 mg/kg bid (n = 18). The total AUC is presented in the right panel (bar graph). Data are the means ± SEM. Statistical analysis was performed using a Student's t test. *P < .05; *** *P* < .001 vs. control. (B) Plasma insulin at indicated time points was assessed in response to oral glucose in the same study. The effect on insulinemia was assessed at individual time points and based on the total AUC from 0 to 120 min calculated with the trapezoidal method. The total AUC is presented in the lower panel (bar graph). Data are the means ± SEM. Statistical analysis was performed using a Student's *t* test. *P < .05; ** *P* < .01 vs. control. (C) Insulinogenic index (ΔI/ΔG), a measure of β‐cell function was calculated. Data are the means ± SEM. Statistical analysis was performed using a Mann‐Whitney U test. *** *P* < .001 vs. control

We first examined the impact imeglimin might have on the general health and metabolic features of ZDF rats. Frequent examination indicated that tolerability to treatment with the compound was indistinguishable from vehicle control—including animal mobility, behaviour and appearance. Compared to vehicle‐treated animals, chronic oral imeglimin treatment (150 mg/kg *bid*. for 5 weeks) also did not affect mean body weight of ZDF male rats (Figure 2). Similarly, the weight of the pancreas, liver and heart was not influenced by imeglimin (data not shown). Basal plasma glucose levels in 3‐hour fasted animals were not significantly reduced after imeglimin treatment for 5 weeks (−7%, NS). The onset of diabetes occurred near the initiation of the treatment period (average glucose levels were 5.75 mM in 7‐week‐old rats) and this model is known to have an extreme phenotype with rapidly progressive hyperglycaemia due to marked β‐cell loss.^19^ However, glucose excursion in response to OGTT in fasted ZDF rats was significantly lower in imeglimin‐treated animals compared with control animals (Figure 3A). Indeed, 5 weeks of imeglimin treatment lowered the total AUC_0‐120_ for plasma glucose by −15%, *P* < .001 (Figure 3A) and the incremental AUC_0‐120 min_ by −33% (*P* < .01) compared to the vehicle‐treated group. This imeglimin‐related improvement in glucose tolerance was directly linked to a concomitant increase in plasma insulin (total AUC_0‐120 min_ + 83%, *P* < .05); however, the incremental change in insulin AUC_0‐120 min_ (+77%) did not achieve statistical significance (Figure 3B). Consequently, the insulinogenic index ΔI_0‐120 min_/ΔG_0‐120 min_ was substantially greater in imeglimin‐treated rats (+165%, *P* < .01) (Figure 3C). Together, these results indicate that chronic imeglimin treatment improves glucose tolerance and amplifies glucose‐stimulated insulin secretion in this extreme male ZDF rat model of T2D with marked hyperglycaemia (20.1 mmol/L).

### 3.2 Imeglimin increases pancreatic insulin content in ZDF rats

**FIGURE 4:**
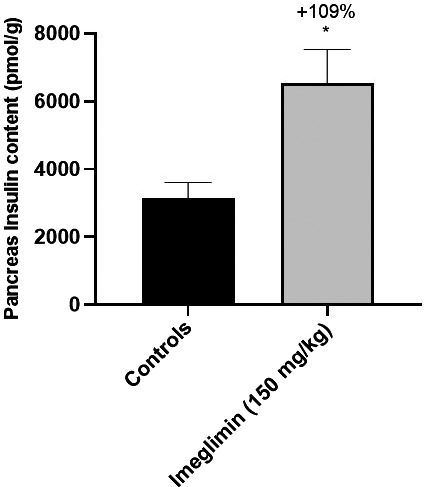
Effect of imeglimin on Pancreatic Insulin Content in ZDF Rats. Data are expressed as mean ± SEM values. Controls n = 4; Imeglimin n = 10. Statistical analysis was performed using a Student's *t* test. * *P* < .05 vs. control

In addition to the aforementioned improvement in insulin secretion in response to glucose challenges, we observed that 35 days of imeglimin treatment resulted in a 72% higher basal insulinemia in 3‐hour fasted ZDF rats compared to control animals (1058 ± 182 *vs*. 613 ± 82 pmol/L, *P* < .05), further suggesting an imeglimin‐mediated improvement of β‐cell function and/or a preservation of β‐cell mass in ZDF rats. Based on these findings, we sought to determine whether chronic imeglimin treatment might also influence pancreatic insulin content. Although mean total pancreas weights were unaffected (−8%, NS), the insulin content per gram of pancreas tissue was increased + 109% in imeglimin‐treated animals compared to the control group (6540 ± 988 vs. 3136 ± 485 pmol/g, *P* < .05) (Figure 4), thus suggesting that imeglimin mitigates the progressive decline of β‐cell mass inherent in the ZDF model.

#### 3.2.1 Imeglimin attenuates loss of β‐cell mass in ZDF rats

**FIGURE 5:**
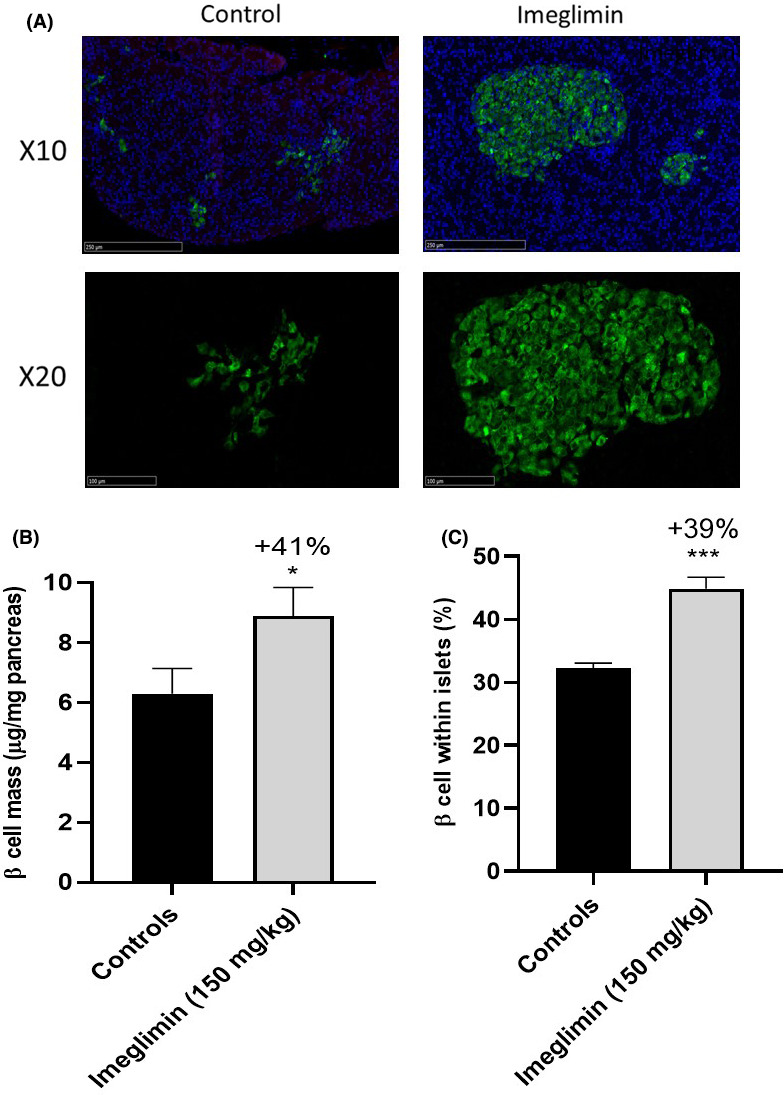
Effect of imeglimin on β‐cell Mass. (A) Representative images of pancreatic islets from ZDF rats after 5 wk of treatment with imeglimin or vehicle. Slices were immunostained with guinea pig anti‐insulin antibody coupled with FITC (green) for analysis of pancreatic islet morphology; blue staining is nucleus labelling with DAPI. The upper panels and the lower panels correspond to X10 and X20 magnification, respectively, of selected areas of pancreas sections. (B) Quantitation of β‐cell Mass. Results are mean ± SEM. Controls n = 4, imeglimin n = 4 (6‐8 observations per rat in each group). Statistical analysis was performed using a Student's *t* test. * *P* < .05 vs. control. (C) Effect of imeglimin on the proportion of β‐cells within islets. Total cells (number of DAPI stained cells) and β‐cells (number of cells staining positive for insulin) were counted. Results are mean ± SEM. Controls n = 4, imeglimin n = 4 (8 to 10 observations per rat in each group). Student's *t* test. ***P < .001 vs control

In the second cohort of animals, we then analysed tissue sections to survey general islet morphology and quantify β‐cell mass and gain further insights into how imeglimin might augment pancreatic insulin content and improve glucose‐stimulated insulin secretion. Insulin staining of pancreatic slices collected from 12‐week‐old ZDF rats (age at the end of the study), which were or were not subjected to 35 days of imeglimin treatment, unveiled qualitative and quantitative differences. As depicted in Figure 5, control ZDF animals predictably presented with overtly abnormal, disrupted pancreatic islet architecture where cells appeared poorly organized and more diffusely aggregated. In contrast, many of the islets derived from imeglimin‐treated rats retained a more normal appearance with rounded, more uniform shape and densely aggregated cells covering a larger surface area. Quantitation of insulin‐positive cells throughout the pancreas revealed a 41% greater β‐cell mass in imeglimin‐treated compared to control rats (8.88 ± 0.96 *vs*. 6.30 ± 0.84 μg/mg, *P* < .05) (Figure 5B). As expected, similar results were obtained when images were analysed to determine the proportion of β‐cells within islets. Here, imeglimin treatment resulted in a 39% (*P* < .001) increase compared to controls (Figure 5C).

#### 3.2.2 Imeglimin alters the balance between β‐cell apoptosis and proliferation

**FIGURE 6:**
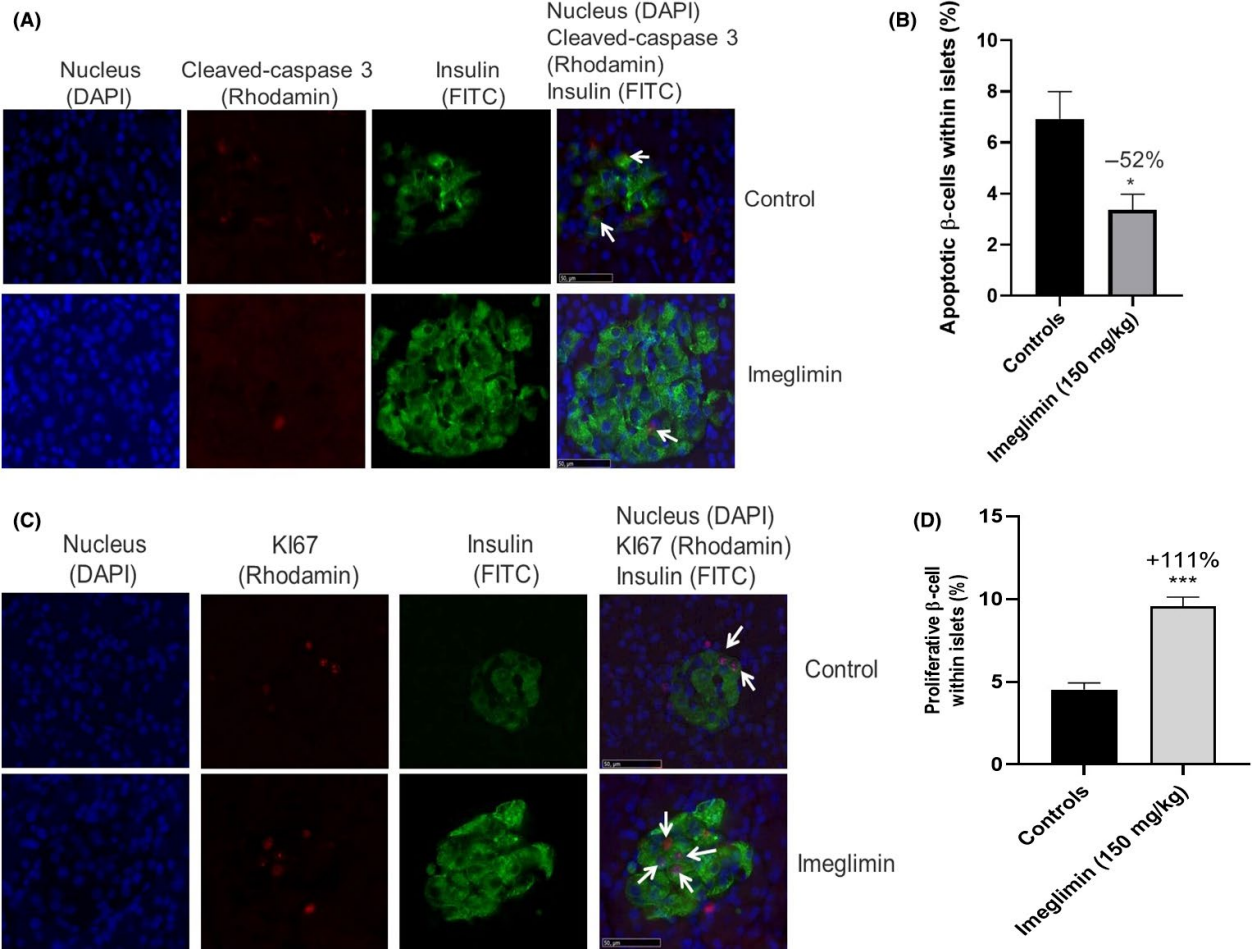
Effect of imeglimin on the Balance Between Apoptosis and Proliferation. (A) Effect of imeglimin on β‐cell apoptosis within islets. Islets from vehicle‐ vs. imeglimin‐treated rats were visualized after immunostaining with a polyclonal rabbit anti‐caspase‐3 antibody (Rhodamine, red) and a polyclonal guinea pig anti‐insulin antibody (FITC, green); double‐stained cells (apoptotic β‐cells) were also visualized (examples noted via white arrows). Nuclei were labelled with DAPI (Blue) (X40 magnification). (B) Quantitation of the proportion of apoptotic β‐cells within islets—islets from control (black) or imeglimin‐treated (grey) rats were double immunostained with anti‐caspase‐3 antibody and an anti‐insulin antibody for β‐cell staining. Nuclei were also labelled with DAPI (Blue). Results are mean ± SEM. Controls n = 4; imeglimin n = 4 (8 to 10 observations per rat in each group). Statistical analysis was performed using a Student's *t* test * *P* < .05 vs. control. (C) Effect of imeglimin on β‐cell proliferation within islets. Islets from vehicle‐ vs. imeglimin‐treated rats were visualized after double immunostaining with a rabbit anti‐Ki67 antibody (Rhodamine, red) and a polyclonal guinea pig anti‐insulin antibody (FITC, green). Nuclei were labelled with DAPI (Blue). Examples of double‐stained proliferating β‐cells are denoted with white arrows. Representative images from the study are shown here (X40 magnification). (D) Effect of imeglimin on the proportion of proliferating β‐cells within islets. Islets from control (black) or imeglimin‐treated (grey) rats were stained as described in A and results were quantitated. Results are mean ± SEM. Controls n = 4; imeglimin n = 4 (5‐6 observations per rat in each group). Statistical analysis was performed using Student's *t* test. *** *P* < .001 vs. control

To better appreciate the mechanism by which imeglimin preserves pancreatic β‐cell mass in ZDF rats, we compared the proportion of apoptotic versus proliferative β‐cells between groups. Apoptotic β‐cells were identified via the dual labelling of insulin and activated caspase‐3, a central player in the apoptosis pathway (Figure 6). We found that 35 days of imeglimin treatment lessened the proportion of apoptotic β‐cells by 52% compared to control ZDF rats (3.36 ± 0.62 *vs*. 6.93 ± 1.06%, respectively; *P* < .05 (Figure 6B)). Interestingly, imeglimin also appeared to somewhat protect insulin‐negative endocrine cells from apoptosis. Thus, the overall proportion of apoptotic endocrine cells (which are mainly beta‐cells) was reduced by 37% in imeglimin‐treated compared to control rats (3.56 ± 0.58 vs. 5.69 ± 0.73%, *P* < .05; data not shown). Remarkably, dual immunostaining for insulin with the proliferative marker Ki67 revealed that imeglimin more than doubled (9.55 ± 0.96 *vs*. 4.52 ± 0.63%, *P* < .001) the proportion of multiplying β‐cells compared to controls (Figure 6C, D). Therefore, imeglimin's effect to maintain greater β‐cell mass in ZDF rats appears to occur via a shift in turnover reflected by reduced apoptosis and increased proliferation.

#### 3.2.3 Imeglimin has no significant effect on β‐cell neogenesis in ZDF rats

**FIGURE 7:**
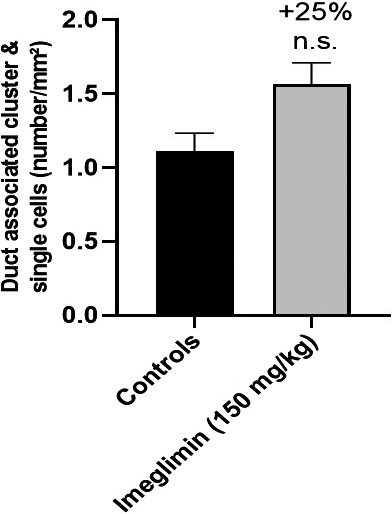
Effect of imeglimin on β‐cell Neogenesis in Pancreases of ZDF rats. Sections were immunostained with a monoclonal rabbit anti‐CK20 antibody (red) and a polyclonal guinea pig anti‐insulin antibody (green). β‐cell neogenesis was evaluated and quantitated based on counting the number of insulin‐positive single cells and small clusters (2‐15 cells) adjacent to CK20‐stained pancreatic ducts. Nuclei were labelled with DAPI. Results are mean ± SEM. Controls n = 4 (20 observations per rat), imeglimin n = 4 (19 observations per rat). Statistical analysis was performed using a Mann‐Whitney test

We next evaluated whether chronic imeglimin treatment fosters β‐cell neogenesis from exocrine duct cells as a potential contributor to β‐cell mass preservation in ZDF rats. Towards this end, we quantified the relative proportion of insulin‐positive single cells or small clusters co‐localizing with, budding from, or in the immediate vicinity (less than 50 μm) of, cytokeratin 20‐positive duct precursor cells. A summary of the quantification of these data are presented in Figure 7; imeglimin produced a nonsignificant trend towards greater β‐cell neogenesis with 21 and 28% mean increases in duct‐associated insulin‐positive single cells and clusters compared to control, respectively.

#### 3.2.4 Imeglimin moderates α‐cell proliferation in ZDF rats

**FIGURE 8:**
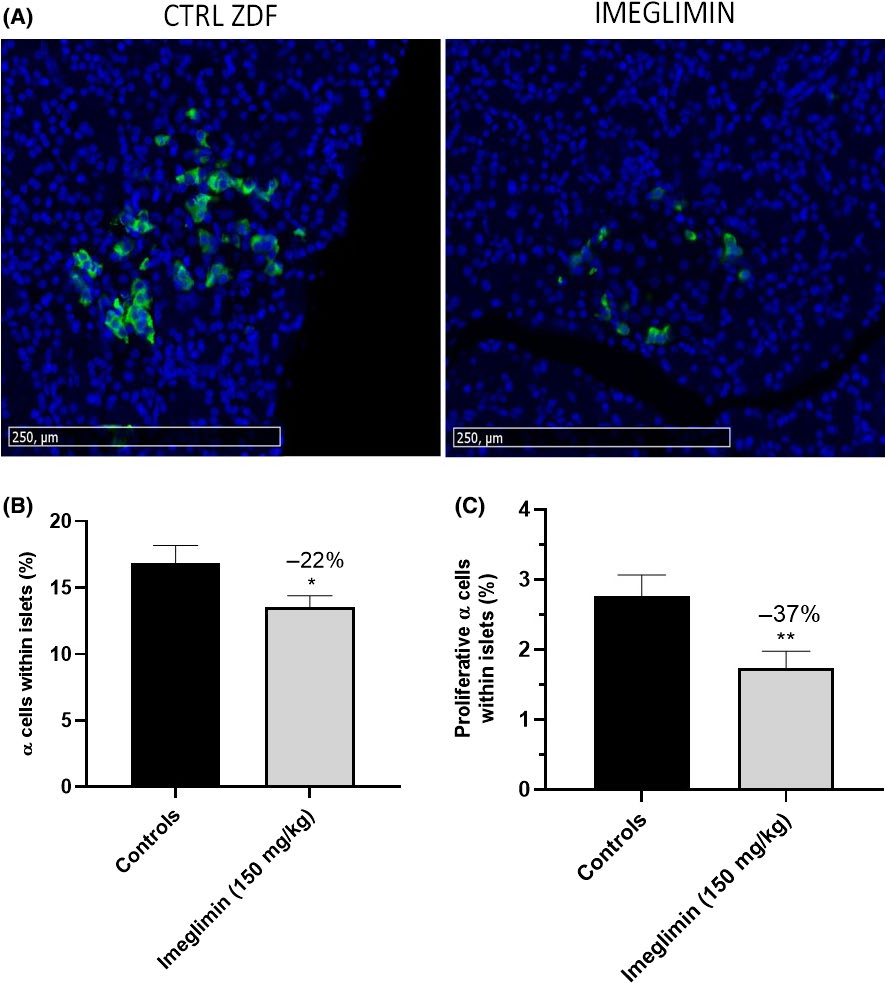
Effect of imeglimin on Pancreatic α‐cells. (A) Glucagon staining of individual islets. Representative images (15.5X magnification) are shown. Pancreas sections were immunostained with a mouse antiglucagon antibody for α‐cells and an anti‐mouse secondary antibody coupled with FITC (green). Nuclei were labelled with DAPI (Blue). (B) Effect of imeglimin on the proportion of α‐cells within islets. Total islet cells (number of DAPI‐stained cells associated with islet structures) and α‐cells (number of cells associated with glucagon labelling) were counted. Results are mean ± SEM. Controls n = 4 (18‐21 observation per rat), imeglimin n = 4 (19‐24 observations per rat). Statistical analysis was performed using a Student's *t* test. *P < .05 vs control. (C) Effect of imeglimin on α‐cell proliferation. Quantitation of α ‐cell proliferation was assessed using pancreas slices with double immunostaining with a rabbit anti‐Ki67 antibody coupled with an Alexa Fluor secondary antibody (red) and a monoclonal mouse antiglucagon antibody coupled with an FITC secondary antibody (green). Nuclei were labelled with DAPI (Blue). Controls n = 4 (17 to 23 observation per rat) and imeglimin n = 4 (17 to 23 observations per rat). Statistical analysis was performed using a Student's *t* test. ** *P* < .01 vs control

As hyperglucagonemia also contributes to T2D pathophysiology, we assayed the impact of chronic imeglimin treatment on glucagon‐producing pancreatic α cells in ZDF rats. As shown in Figure 8, the proportion of glucagon‐positive cells per islet was reduced by 22% in imeglimin‐ compared to vehicle‐treated ZDF rats (13.4 ± 0.6 *vs*. 17.1 ± 1.0%, *P* < .05). Increases in the number of β‐cells per islet could contribute to this effect. However, five weeks of treatment with imeglimin was also observed to produce a trend towards a decrease in total pancreas α‐cell mass (−21%, NS—data not shown). In alignment with this result, we also found that glucagon/Ki67 double‐stained cells were lowered by 37% in islets from imeglimin‐treated rats (1.74 ± 0.24 *vs*. 2.76 ± 0.30%, *P* < .01) indicating reduced α‐cell proliferation (Figure 8C).

## 4 DISCUSSION

Type 2 diabetes (T2D) pathophysiology is linked to compromised insulin secretion caused by the progressive dysfunction and loss of pancreatic β‐cells.^20^ Since Lerner and Porte first provided compelling evidence pointing towards defective insulin storage and/or secretion in patients with T2D,^21^ β‐cell failure is acknowledged as the primary underlying cause of overt hyperglycaemia and T2D progression,^20^thus, there is now little doubt deficient insulin secretion in T2D results both from β‐cell dysfunction and β‐cell death.^22^, ^23^ Hence, restoration of β‐cell function and preservation of β‐cell mass have obvious therapeutic potential.

Representative animal models that recapitulate the progressive demise of β‐cells are therefore useful tools. The obese hyperglycaemic ZDF male rat model is well suited as it mimics key features of T2D including fasting hyperglycaemia, glucose intolerance, insulin resistance and marked, progressive, dysfunction and apoptosis of pancreatic β‐cells.^19^, ^24^, ^25^, ^26^ In this study, we used ZDF rats to successfully address the hypothesis that imeglimin, a novel therapeutic agent, could ameliorate the loss of insulin secretory capacity and β‐cell mass in the context of severe diabetes. In addition, we unveiled an additional unexpected effect of imeglimin to reduce islet α cells (via reduced proliferation) in this model.

We first observed that 5 weeks of chronic imeglimin treatment resulted in improved glucose tolerance with enhanced insulinemia in response to glucose challenges in animals that had achieved 12 weeks of age at the time they were assessed. The combination of these effects produced substantial increases in the insulinogenic index (+165%). Somewhat surprisingly, basal hyperglycaemia was unaffected by imeglimin despite an improvement of glucose tolerance. This underscores the extreme nature of this model, which cannot be fully overcome by a ≈2‐fold increase in insulinemia. We also hypothesize that an effect on basal glycemia could have potentially occurred if treatment had started earlier, before the onset of diabetes. Nevertheless, these findings are consistent with several prior studies reporting imeglimin‐mediated improvements in GSIS in other T2D rodent models.^12^, ^13^, ^14^ Moreover, the current results are consistent with existing clinical data including a clear effect of imeglimin to enhance GSIS in T2D patients during a hyperglycaemic clamp.^11^

The finding of a substantial increase in pancreatic insulin content (+109%), along with increases in basal insulinemia in imeglimin‐treated (vs. vehicle control) ZDF rats, suggested the potential for an effect of imeglimin on β‐cell mass. Although increased insulin content could occur via greater insulin per β‐cell, our additional results support a primary effect on β‐cell mass. Indeed, we demonstrated such an effect by showing a net increase in insulin‐positive cells in pancreas tissue from treated rats (resulting a mean + 41% effect) along with an increase (+39%) in the proportion of β‐cells per islet. Importantly, an improvement in islet morphology was also noted. To gain further insights into the mechanisms involved, we examined indices of β‐cell turnover via immunohistochemistry. The percentage of apoptotic β‐cells was clearly suppressed and a reciprocal increase in β‐cells undergoing proliferation was also found. Having failed to see a significant increase in β‐cells associated with pancreatic ducts, the data suggest that imeglimin treatment may preferentially induce proliferation of existing cells rather than affecting neogenesis from ductal precursors. Nonetheless, as the assessment of neogenesis was solely performed at study completion, a prior effect of imeglimin at earlier stages cannot be ruled out. Considering that pancreatic β‐cells are long‐lived and mostly senescent in adult humans,^27^, ^28^ the translational potential of an imeglimin‐induced effect to induce proliferation of β‐cells is quite uncertain. In contrast, the potential for β‐cell preservation mediated by reduced apoptosis may be greater since diabetes‐associated reductions in β‐cell mass are thought to primarily result from apoptosis in both ZDF rats as well as in patients with T2D.^5^, ^19^, ^29^

Several prior lines of evidence have suggested the potential for imeglimin to preserve functional β‐cell mass. Firstly, it is clear that imeglimin has a direct (and acute) effect to modulate islet β‐cell function manifested by an increase in GSIS that is similar to GLP‐1 but involving a distinct non‐cAMP dependent pathway.^12^, ^13^, ^30^ Moreover, the molecule was shown to prevent the death of cultured rat β cells and INS‐1 cells when exposed to pro‐inflammatory cytokines and high glucose, respectively.^12^ Similarly, a preliminary report revealed that imeglimin prevents β‐cell apoptosis induced by high glucose in both rat and human isolated islets.^31^ These findings and the current results beg the following question: What mechanism(s) may underlie the ability of imeglimin to reduce β‐cell apoptosis and preserve mass?

Although the mechanism(s) for the chronic β‐cell protective effect shown here were not interrogated, there are some intriguing possibilities. Imeglimin is known to modulate mitochondrial function; mitochondrial dysfunction has a clear role in contributing to T2D pathophysiology ^16^, ^17^, ^18^—including defects described in β‐cells derived from both ZDF rats and T2D patients.^16^, ^18^, ^32^, ^33^, ^34^ Imeglimin was also shown to prevent opening of the mitochondrial permeability transition pore (PTP). This has been demonstrated in endothelial cells ^15^ and is a known driver of apoptotic cell death that occurs as a consequence of mitochondrial dysfunction.^35^, ^36^

An additional related effect of imeglimin involves its ability—in isolated islets—to increase cellular NAD^+^ generation from nicotinamide via NAMPT in the ‘salvage’ synthetic pathway.^30^ Increases in the cellular NAD^+^ pool can, in turn, enhance GSIS via the generation of second messengers that augment Ca^++^ mobilization in response to glucose.^37^ Obviously, NAD^+^ (via conversion to NADH) is also a critical co‐factor for the mitochondrial respiratory chain.^38^ In relation to longer term effects to preserve β‐cell mass, it has been reported that mitochondrial NAD^+^ levels regulate apoptosis,^39^ that depletion via NAMPT inhibition provokes apoptosis ^40^ and that exogenous NAD^+^ protects cells from apoptosis in response to several stressors.^41^ In addition, exogenous nicotinamide can induce differentiation and maturation of human foetal pancreatic islet cells.^42^ In an additional preliminary experiment, cellular NAD^+^ content was increased by 28% (*P* < .05) in islets isolated from ZDF rats treated for 5 weeks with imeglimin (150mg/kg bid) and then incubated in vitro with imeglimin (100 µM) for 20 minutes vs. islets from imeglimin‐treated rats without in vitro exposure to imeglimin. Therefore, it is intriguing to consider that imeglimin’s acute effect to increase cellular NAD^+^ in islets may contribute to the longer term reduction in apoptosis and retention of β‐cell mass.

Finally, it is interesting to further consider the implications of the more modest potential effect of imeglimin to also reduce islet‐associated α cells, which was associated with a demonstrable reduction in α‐cell proliferation. Unfortunately, the present study was limited by the absence of circulating glucagon measurements. Although α‐cell mass is not known to be increased in ZDF rats,^43^ a shift in the balance of α vs. β‐cells may still contribute to hyperglycaemia in this model. It is also possible that imeglimin mediates some degree of trans‐differentiation from α to β‐cells. Importantly, glucagon contributes to both fasting as well as postprandial hyperglycaemia in human T2D.^44^ Fasting glucagon levels were unaffected in patients after short‐term (7 day) treatment with imeglimin,^11^however, this observation does not exclude the potential for longer term effects on α‐cell mass and glucagon tone. We also note than any potential decrease in glucagon in humans is not of significant magnitude to potentiate hypoglycaemia. Clinical data obtained to date—including co‐administration with insulin in patients (TIMES 3 Phase 3 trial, Poxel SA, unpublished)—have so far not shown significant increases in incidence or severity of hypoglycaemic events.

We did not assess insulin sensitivity in these experiments; although this represents an additional limitation, previous studies have shown improvements in insulin sensitivity in appropriate rodent models.^14^

Collectively, the results of these experiments demonstrate that chronic treatment with imeglimin ameliorates glucose intolerance and augments insulinemia in an extreme model of T2D; increases in β‐cell mass, resulting from a combined effect to suppress apoptosis and increase proliferation, were documented. These observations lead to an intriguing hypothesis—that imeglimin may mediate disease‐modifying effects to preserve or augment β‐cell mass in humans. Such actions could potentially lead to greater durability in the context of established T2D and might also be considered as a means of preventing the transition from impaired glucose tolerance to T2D.

## CONFLICT OF INTEREST

This work was funded by Poxel SA as part of the development programme for imeglimin. SHB, DEM and SB are employees of Poxel and stockholders.

## AUTHOR CONTRIBUTIONS

SHB, MK and SB designed and implemented and/or supervised experiments described herein. DEM and all other authors engaged in data analysis and interpretation. All authors contributed to and reviewed the manuscript.

## Data Availability

The data that support the findings of this study are available from the corresponding author upon reasonable request.
